# Microsurgical Outcomes of Third-Ventricle Colloid Cysts Treated via an Interhemispheric Transcallosal Approach: A Single-Center Retrospective Series

**DOI:** 10.3390/brainsci16101101

**Published:** 2026-10-13

**Authors:** Abdulkadir Kankılıç, İbrahim Başar, Ferhat Kurtoğlu, Ali Fahir Özer

**Affiliations:** 1 Department of Neurosurgery, Faculty of Medicine, Harran University, 63300 Şanlıurfa, Türkiye; 2 Department of Neurosurgery, Faculty of Medicine, Dicle University, 21280 Diyarbakır, Türkiye; 3 Department of Neurosurgery, Koç University Hospital, 34010 Zeytinburnu, Türkiye

**Keywords:** third-ventricle colloid cyst, interhemispheric transcallosal approach, microsurgical resection, hydrocephalus, Karnofsky Performance Status, gross-total resection

## Abstract

Background/Objectives: Third-ventricle colloid cysts are benign but can cause obstructive hydrocephalus and sudden deterioration, and the role of microsurgical transcallosal resection in the endoscopic era remains debated. We evaluated clinical, radiological, and functional outcomes of microscope-assisted interhemispheric transcallosal resection and the effect of preoperative hydrocephalus on recovery. Methods: This retrospective, observational, single-center study analyzed 30 patients with symptomatic, histopathologically confirmed colloid cysts operated on between June 2015 and February 2021, divided into hydrocephalus (*n* = 16) and non-hydrocephalus (*n* = 14) groups. Functional outcomes were assessed using the Karnofsky Performance Status (KPS) scale. Results: Mean KPS improved from 65.3 ± 21.0 to 92.0 ± 11.0 (*p* < 0.001). Patients with hydrocephalus had lower preoperative Glasgow Coma Scale (GCS; *p* = 0.022) and KPS (*p* = 0.048) scores, comparable postoperative KPS (*p* = 0.208), and greater KPS improvement (*p* = 0.012). Four patients (13.3%) needed external ventricular drainage; none required a permanent shunt. Four patients (13.3%) developed new persistent neurological events (seizures, *n* = 3; memory impairment, *n* = 1). No recurrence occurred over a median follow-up of 7.0 years. Conclusions: Microscope-assisted interhemispheric transcallosal microsurgical resection was associated with significant KPS improvement, no permanent shunt requirement, and no recurrence. Despite the increasing use of endoscopic techniques, microsurgical approaches remain valuable due to direct visualization, bimanual dissection, and the potential for durable gross-total resection; however, neurocognitive outcomes were not assessed, and prospective studies are needed.

## 1. Introduction

Rare and histologically benign, third-ventricle colloid cysts typically develop close to the foramen of Monro [1,2]. Their placement makes them clinically significant despite their benign pathology since blockage of cerebrospinal fluid (CSF) routes can result in hydrocephalus, elevated intracranial pressure, rapid neurological impairment, or in rare cases, death [3,4]. Due to their low occurrence and the small size of most documented series, it is challenging to describe their natural history; nonetheless, active treatment is usually necessary for symptomatic lesions and those linked to hydrocephalus [1]. Surgical intervention is required for all patients with symptomatic colloid cysts and for asymptomatic patients presenting with high-risk clinical or radiological features [5,6].

Ventricular blockage, cyst size, radiological risk factors, and symptom status all affect management. The Colloid Cyst Risk Score (CCRS), which takes into account factors like age, headache, cyst diameter, fluid-attenuated inversion recovery (FLAIR) hyperintensity, and cyst position inside the third ventricle, was created to detect symptomatic patients and stratify the risk of hydrocephalus [6,7].

Surgery remains the primary and most effective treatment for symptomatic third-ventricle colloid cysts, although the optimal surgical strategy remains controversial [8,9]. The main advantage of surgery is that it definitively removes the obstructing lesion, restores CSF flow, and markedly reduces the risk of acute hydrocephalus and sudden neurological deterioration; in large surgical series, resection has been followed by relief of obstructive symptoms and favorable clinical outcomes [10,11,12], whereas CSF diversion alone leaves the underlying lesion untreated. While microsurgical resection has been linked in systematic reviews and meta-analyses to higher rates of gross-total resection and reduced recurrence, endoscopic resection is less invasive and may be associated with lower perioperative morbidity [8,13]. Although endoscopic surgery offers the advantages of a less invasive approach and a potentially more favorable perioperative morbidity profile, it also has recognized disadvantages. The narrow working channel and the restricted maneuverability of endoscopic instruments limit bimanual dissection and may hamper control of bleeding and complete detachment of the cyst wall from the fornices, choroid plexus, and venous structures. Consequently, concerns regarding incomplete cyst wall removal and long-term recurrence persist. A recent systematic review of 34 studies involving 1123 patients who underwent endoscopic resection reported postoperative cyst remnants in 17.1% of patients, recurrence or regrowth in 3.7%, and reoperation in 2.9%, with a mean follow-up of 46.3 months [14]. Conversely, microsurgical transcallosal resection is more invasive and has been associated with longer operating times and hospital stays and with higher overall morbidity [8,13]. When choosing a course of treatment for individuals with obstructive hydrocephalus or decreased neurological state, this trade-off is very important.

Although stereotactic aspiration of colloid cysts has been used historically, current surgical management generally involves cyst decompression and excision of the cyst wall while minimizing injury to adjacent neurovascular structures, particularly the fornix, deep intraventricular veins, and the ependymal lining of the third ventricle [5,15,16].

Endoscopic surgery and microsurgical transcallosal surgery can both provide excellent surgical outcomes in the treatment of colloid cysts; however, an important limitation of endoscopic surgery is the lower likelihood of achieving complete removal of the cyst wall [13,14]. Given the slow growth tendency of colloid cysts, it remains difficult to make definitive conclusions regarding recurrence rates based on the relatively short follow-up periods reported in most studies in the literature [17].

The interhemispheric transcallosal approach is a well-established microsurgical route to anterior third-ventricle colloid cysts. It provides a direct midline corridor to the lateral ventricle and foramen of Monro while avoiding cortical transgression and allows bimanual microsurgical dissection of the cyst wall from the forniceal, venous, and choroidal structures [11,12]. Large transcallosal series, including the 134-patient experience reported by Hernesniemi and colleagues [10] and the 297-patient experience reported by Nair and colleagues [12], support the effectiveness of this approach when meticulous attention is paid to the fornices, deep venous structures, and callosal entry. Nevertheless, seizure, memory impairment, venous injury, and other neurological events remain important concerns after transcallosal surgery [10,11,18].

Because colloid cysts grow slowly, recurrence can only be recognized after prolonged follow-up, and because obstructive hydrocephalus is the main determinant of clinical severity and urgency, long-term data on the durability of microsurgical resection and on the influence of hydrocephalus on recovery are of particular interest. In this study, we evaluated clinical, radiological, and functional outcomes in 30 patients with third-ventricle colloid cysts treated using a microscope-assisted interhemispheric transcallosal approach. We specifically focused on postoperative functional recovery and the impact of preoperative hydrocephalus on baseline clinical status and postoperative outcome. We hypothesized that preoperative hydrocephalus would be associated with a poorer baseline clinical and functional status, but that definitive microsurgical relief of the obstruction would lead to comparable postoperative functional outcomes, without permanent shunt dependency or recurrence during long-term follow-up.

## 2. Methods

### 2.1. Ethical Statement

The study was conducted in accordance with the Declaration of Helsinki. The study protocol was reviewed and approved by the Ethics Committee for Noninterventional Studies of Dicle University Faculty of Medicine (decision date: 15 April 2026; decision no.: 205). The Ethics Committee approved the retrospective use of previously collected clinical and radiological data and waived the requirement for informed consent because of the retrospective design. Patient data were anonymized before analysis, and patient confidentiality was protected in accordance with institutional data-protection regulations.

### 2.2. Study Design and Patient Selection

This was a retrospective, observational, single-arm, single-center cohort study conducted at a tertiary neurosurgical center. The study was non-randomized and unblinded, and no experimental intervention was applied. Between June 2015 and February 2021, 30 patients underwent surgery for third-ventricle colloid cysts at our center. All patients underwent microscope-assisted interhemispheric transcallosal microsurgical excision, and the diagnosis of colloid cyst was confirmed histopathologically. Total excision of the cyst was achieved in all patients, with no residual lesion identified postoperatively. Patients were eligible for inclusion if they underwent total excision of a third-ventricle colloid cyst through the interhemispheric transcallosal approach, had histopathological confirmation of the diagnosis, and had a minimum postoperative follow-up of 5 years. Patients with less than 5 years of postoperative follow-up were excluded. No additional clinical eligibility criteria, such as specific laboratory thresholds, age restrictions, or anesthetic-risk cut-offs, were applied. Patients requiring perioperative external ventricular drainage (EVD) were also included, provided that total cyst excision was achieved through the interhemispheric transcallosal microsurgical approach.

Patients who underwent primary endoscopic, transcortical, or stereotactic treatment, those managed conservatively, or those without histopathological confirmation of a colloid cyst were excluded. Radiological archives, operative reports, institutional medical records, and follow-up files were retrospectively reviewed. Postoperative radiological assessment included brain computed tomography (CT) within the first postoperative hour and contrast-enhanced brain magnetic resonance imaging (MRI) within the first 24 h after surgery to assess the immediate postoperative status and confirm gross-total cyst excision. Subsequent MRI evaluations were performed at 1, 3, and 6 months and 1 year postoperatively. After the first postoperative year, MRI follow-up was performed at intervals of at least 1 year. The final cohort consisted of 30 patients aged 10–55 years, all of whom had a minimum postoperative follow-up of 5 years.

Clinical and radiological data obtained during routine evaluations were systematically recorded in our institutional database as part of standard patient care. After ethics approval (Section 2.1), these previously collected data were retrospectively reviewed and analyzed for the purposes of the present study. Radiological archives, operative reports, institutional medical records, and follow-up files were retrospectively reviewed.

### 2.3. Data Collection, Clinical and Radiological Variables

Age, sex, presenting symptoms, long-term symptom history, duration of symptoms, preoperative Glasgow Coma Scale (GCS) score, and preoperative Karnofsky Performance Status (KPS) score were among the clinical and demographic factors. Maximum cyst diameter, hydrocephalus, T1-weighted magnetic resonance imaging (MRI) signal, T2-weighted MRI signal relative to CSF, FLAIR signal, contrast enhancement, and computed tomography (CT) density were among the radiological factors. The biggest detectable diameter on preoperative imaging was used to quantify cyst size. Ventricular swelling caused by blockage at the level of the third ventricle or the foramen of Monro was described as hydrocephalus. Hydrocephalus was defined based on an increased Evans index, effacement of the cerebral sulci, and changes in cerebral parenchymal density on CT suggestive of ventricular dilatation. An increased Evans index alone was not considered sufficient for the diagnosis of hydrocephalus.

The GCS [19] was used to grade the level of consciousness at admission, and the KPS [20,21,22] was used to assess functional status. The GCS ranges from 3 to 15, and the KPS is an ordinal scale ranging from 0 (death) to 100 (normal activity without symptoms) in 10-point steps. Both are standard, widely used instruments; the KPS has been evaluated for reliability and validity mainly in oncology populations [21,22], whereas neither scale has been specifically validated in patients with colloid cysts. GCS and KPS scores were retrospectively derived from the clinical records documented at admission and during follow-up visits.

EVD, the need for a permanent shunt, infection, recurrence, postoperative headache status, postoperative KPS, and postoperative neurological sequelae were among the perioperative and postoperative factors. New postoperative neurological events were defined as neurological deficits or symptoms not present before surgery. Pre-existing neurological deficits were recorded separately and assessed for persistence at the last follow-up. New postoperative events were classified as transient if they completely resolved by the last follow-up and as persistent if they remained present at the last follow-up. Events were categorized by type as seizure, motor deficit, visual deficit, or memory impairment. A radiological reappearance or enlargement of a residual or recurrent colloid cyst during the follow-up period was considered recurrence.

Postoperative radiological assessment was performed with a brain CT scan within the first postoperative hour and contrast-enhanced brain MRI within the first 24 h after surgery to assess the immediate postoperative status and confirm gross-total cyst excision. Subsequent MRI evaluations were performed at 1, 3, and 6 months and 1 year postoperatively. After the first postoperative year, MRI follow-up was performed at intervals of at least 1 year. The variables and the time points at which they were assessed are summarized in Table 1. Pre- and postoperative imaging studies had been routinely obtained as part of clinical care and were interpreted by an experienced neuroradiologist who was not a member of the study team and was unaware of the present study. The corresponding radiological reports were generated independently of the study. For the present retrospective analysis, the study surgical team reviewed the available imaging studies together with the corresponding radiological reports. Thus, the radiological reports were independently generated, whereas the study-specific retrospective imaging review was performed by the surgical team.

### 2.4. Anesthetic Management

All procedures were performed under general anesthesia with propofol and midazolam used for induction and sevoflurane used for maintenance, with standard intraoperative monitoring including invasive arterial pressure monitoring. Intracranial pressure and brain relaxation were managed with appropriate head and neck positioning, leaving sufficient space around the chin and neck to facilitate venous return, and with mannitol administration in all patients. Dexamethasone was administered in all patients, and sodium replacement was performed in patients with hyponatremia to prevent or treat cerebral edema and increased intracranial pressure. Perioperative antiepileptic prophylaxis was not routinely administered; corticosteroids were administered in all patients; and American Society of Anesthesiologists (ASA) physical status was assessed routinely by the anesthesiology team [23]. However, ASA classification was not included as a study variable or in the statistical analysis. Throughout the procedures, anesthetic and hemodynamic management was performed by the anesthesiology team with the aim of maintaining adequate cerebral perfusion and preventing or minimizing cerebral edema. Anesthetic variables were not included in the statistical analysis because anesthetic management may influence early postoperative neurological and functional assessment; this is acknowledged as a limitation.

### 2.5. Surgical Technique

All patients underwent microscope-assisted interhemispheric transcallosal resection of the colloid cyst performed by the same surgical team. Under general anesthesia, patients were placed in the supine position with the head secured in a three-pin Mayfield head holder. The head and trunk were positioned in slight flexion while ensuring adequate cerebral venous drainage and avoiding impairment of venous outflow. No head rotation was performed.

A frontal skin flap with an anteriorly directed base was raised, with all margins of the flap remaining within the hair-bearing scalp. A parasagittal frontal craniotomy measuring approximately 5 × 5 cm was then performed, extending approximately 2 cm posterior and 3 cm anterior to the right coronal suture. The craniotomy extended approximately 1.5–2 cm lateral to the midline on the left and 3 cm lateral to the midline on the right. The dura mater was opened in a C-shaped fashion and reflected toward the left over the superior sagittal sinus, providing a perpendicular operative view along the edge of the falx cerebri.

The interhemispheric fissure was microscopically dissected, and bridging veins were preserved whenever possible. The pericallosal arteries and corpus callosum were identified. A limited anterior callosotomy measuring less than 1.5 cm was performed to access the lateral ventricle. During the dissection, the exposed brain tissue was protected with Surgicel to minimize direct mechanical trauma and was frequently moistened with normal saline throughout the procedure to prevent tissue desiccation.

Upon entering the lateral ventricle, the septal vein, thalamostriate vein, choroid plexus, fornix, and foramen of Monro were identified. Depending on the anatomical characteristics and location of the lesion, the cyst was approached through the foramen of Monro or an adjacent safe ventricular corridor. The cyst contents were initially evacuated to achieve internal decompression, followed by careful dissection and complete excision of the cyst wall using sharp microsurgical dissection. The septal and thalamostriate veins, fornices, deep venous structures, and surrounding ventricular anatomy were preserved throughout the procedure. Complete excision of the cyst wall was achieved in all patients.

Hemostasis was achieved under microscopic visualization, followed by standard closure of the dura, bone flap, and scalp.

In patients presenting with rapidly progressive and clinically significant signs of increased intracranial pressure, external ventricular drainage (EVD) was performed as the initial surgical procedure. Definitive microsurgical resection of the colloid cyst was subsequently performed immediately after EVD placement. The EVD was maintained at the level of the patient’s head to allow controlled cerebrospinal fluid drainage. Postoperative imaging was performed to assess cerebrospinal fluid circulation. In the absence of significant postoperative impairment of cerebrospinal fluid circulation, the EVD was clamped on postoperative day 3, and the patient was observed for 24 h. If no deterioration in the Glasgow Coma Scale score or clinical status occurred during the clamping period, the EVD was removed.

The cyst wall and its contents were submitted for histopathological examination in all cases.

### 2.6. Outcomes

Functional recovery, as determined by the difference in KPS between the preoperative and postoperative assessments, was the main endpoint. Preoperative hydrocephalus and baseline clinical severity, postoperative KPS, degree of KPS improvement, EVD necessity, permanent shunt reliance, infection, recurrence, and postoperative neurological complications were among the secondary outcomes. Neurocognitive function was not formally assessed.

Patients were categorized based on whether or not they had radiological hydrocephalus prior to surgery. Because hydrocephalus is a clinically significant indicator of obstructive severity in third-ventricle colloid cysts and can affect neurological status, treatment urgency, the requirement for cerebrospinal fluid diversion, and postoperative recovery, this subgroup analysis was chosen.

### 2.7. Statistical Analysis

Statistical analyses were performed using R software, version 4.5.1 (R Foundation for Statistical Computing, Vienna, Austria) [24]. Continuous variables were presented as mean ± standard deviation (SD) and median with interquartile range (IQR). Categorical variables were reported as frequencies and percentages. Normality was assessed using the Shapiro–Wilk test. Because several variables were not normally distributed and the sample size was limited, non-parametric tests were used.

Preoperative and postoperative KPS scores were compared using the Wilcoxon signed-rank test. Comparisons between patients with and without hydrocephalus were performed using the Mann–Whitney U test for continuous variables and Fisher’s exact test for categorical variables. A two-sided *p* value <0.05 was considered statistically significant. Exact *p* values are reported, except that values below 0.001 are reported as *p* < 0.001. Because the subgroup comparisons were exploratory, no adjustment for multiple comparisons was made. Multivariable regression analysis was not performed because of the limited sample size and risk of overfitting. Missing data were not imputed, and analyses were performed using available cases.

## 3. Results

### 3.1. Patient Characteristics and Clinical Presentation

The study comprised thirty patients who had microsurgical interhemispheric transcallosal excision for colloid cysts in the third ventricle. Seventeen patients (56.7%) were female and 13 (43.3%) were male, and the average age was 30.9 ± 10.6 years. The median cyst diameter was 12.5 mm, while the mean maximum cyst diameter was 14.0 ± 6.0 mm. Of the cohort, 16 patients (53.3%) were found to have radiological hydrocephalus and 14 (46.7%) had no hydrocephalus. Radiological hydrocephalus was identified in 16 patients (53.3%), based on an increased Evans index, effacement of the cerebral sulci, and changes in cerebral parenchymal density on CT suggestive of ventricular dilatation.

ASA physical status was assessed routinely by the anesthesiology team; however, ASA classification was not included as a study variable.

The predominant preoperative presenting symptom was headache in 18 patients, vomiting in 8 patients, and visual impairment and sudden loss of consciousness in two patients each. In addition to the predominant symptoms, signs of increased intracranial pressure were present. Long-standing symptoms were present in all patients (30/30, 100%), with headache being the predominant symptom (27/30, 90.0%). Among patients with an available quantitative symptom duration (*n* = 30), the median duration was 24 months (IQR, 10.5–48 months; range, 1–144 months).

Although individuals who presented with acute clinical deterioration had lower scores, the median preoperative GCS score was 15. Variable signal qualities were shown by preoperative radiological examination. Twelve cysts were hyperintense and eighteen were hypointense on T1-weighted MRI. With the exception of one hyperintense lesion, nearly all lesions on T2-weighted imaging in relation to CSF were hypointense. Twenty-three patients had FLAIR hyperintensity. There was no contrast enhancement in any of the lesions. Twenty cysts were hyperdense, eight were isodense, and two were hypodense on CT (Table 2).

### 3.2. Functional Outcomes

Following surgery, functional status significantly improved. The preoperative KPS had a median of 80.0 and a mean of 65.3 ± 21.0. The mean KPS rose to 92.0 ± 11.0 after surgery, with a median of 90.0. The median KPS improvement was 20.0 points, while the mean improvement was 26.7 ± 19.7 points.

**Figure 1:**
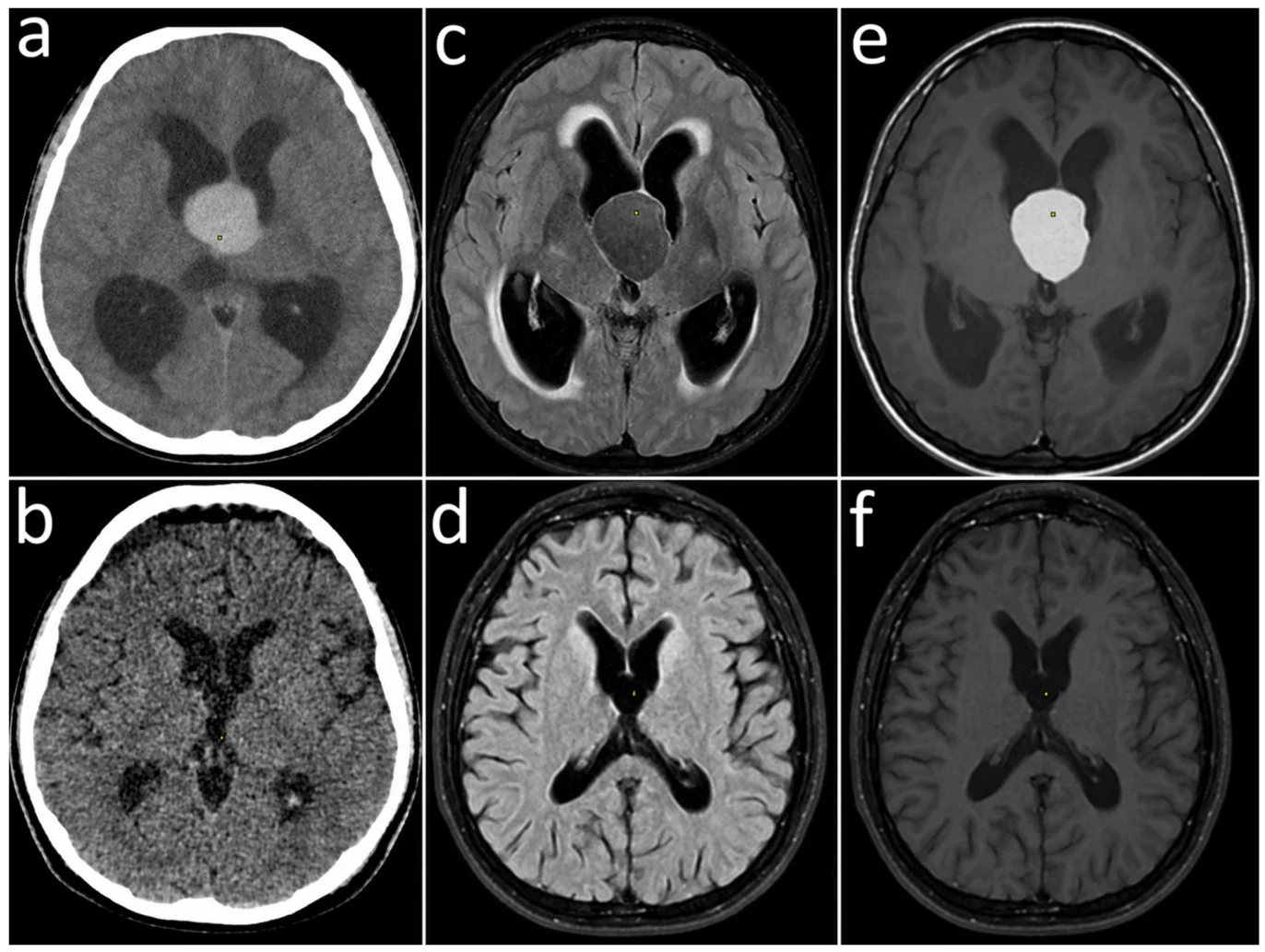
Preoperative and postoperative 5-year follow-up CT and MRI images of a patient. Upper row (**a**,**c**,**e**): preoperative images; lower row (**b**,**d**,**f**): corresponding 5-year follow-up images. Images (**a**,**b**) demonstrate computed tomography (CT) sections. Images (**c**,**d**) correspond to magnetic resonance imaging (MRI) FLAIR sequences, while images (**e**,**f**) correspond to MRI T1-weighted sequences. The preoperative images show a colloid cyst at the foramen of Monro with dilated lateral ventricles; the follow-up images show no residual or recurrent cyst and reduced ventricular size.

A paired analysis utilizing the Wilcoxon signed-rank test revealed a statistically significant rise in KPS from preoperative to postoperative testing (*p* < 0.001). Figure 1 shows preoperative (panels a, c, e) and postoperative 5-year follow-up (panels b, d, f) CT and MRI images of a representative patient demonstrating successful cyst resection and ventricular decompression.

Of the patients whose postoperative headache status could be evaluated, 16 had no headache and 13 had less headache. Due to a poor initial clinical condition, one patient’s postoperative headache status could not be properly examined (this patient is described in Section 3.4).

In patients who experienced a reduction in headache, it was determined that their persistent headache symptoms were not severe enough to interfere with their daily activities or their return to work. In the patient whose postoperative headache could not be assessed, it was observed that the loss of consciousness that had developed in the preoperative period and the severe loss of neurological function at the time of admission to hospital persisted in the postoperative period as permanent motor deficits.

The need for EVD during the acute postoperative period and complications such as infection, as well as the need for a permanent shunt assessed during long-term follow-up, postoperative neurological events, seizures, memory impairment, motor deficits, vision loss and recurrence rates are shown in Table 3.

### 3.3. Hydrocephalus Subgroup Analysis

Patients were stratified according to the presence or absence of preoperative radiological hydrocephalus. Fourteen patients had no hydrocephalus, whereas 16 patients presented with hydrocephalus (Table 4).

Patients with hydrocephalus tended to have larger cysts than those without hydrocephalus, although this difference did not reach statistical significance. The median cyst diameter was 15.0 mm in the hydrocephalus group and 11.0 mm in the non-hydrocephalus group (*p* = 0.076). Age did not differ between the groups (*p* = 0.279).

Hydrocephalus was associated with worse baseline neurological and functional status. Patients with hydrocephalus had significantly lower preoperative GCS scores compared with patients without hydrocephalus (*p* = 0.022). Similarly, preoperative KPS was significantly lower in the hydrocephalus group. The median preoperative KPS was 60.0 in patients with hydrocephalus and 80.0 in those without hydrocephalus (*p* = 0.048).

Despite this worse baseline status, postoperative functional outcomes were comparable between the two groups. Median postoperative KPS was 95.0 in the hydrocephalus group and 90.0 in the non-hydrocephalus group, with no statistically significant difference between groups (*p* = 0.208). KPS improvement was significantly greater among patients with hydrocephalus, with a median improvement of 20.0 points compared with 10.0 points in patients without hydrocephalus (*p* = 0.012). EVD was required only in the hydrocephalus group (0% vs. 25.0%; *p* = 0.103), and the frequencies of neurological events or persistent deficits (14.3% vs. 25.0%; *p* = 0.657) and infection (0% vs. 6.3%; *p* = 1.000) did not differ significantly between the groups.

### 3.4. Postoperative Events, Shunt Requirement, and Recurrence

At the most recent follow-up, six patients had persistent neurological events or deficits. Three patients experienced recurrent seizures that were successfully controlled with antiepileptic medication. One patient had persistent memory impairment characterized by difficulty retaining recent events, which continued to interfere with daily activities at last follow-up. Two patients had persistent motor deficit and visual deficit, respectively; both had presented preoperatively with the same deficits following sudden loss of consciousness and severe neurological deterioration, and these deficits did not resolve postoperatively. No transient neurological events—defined as events that fully resolved before last follow-up—were identified in this cohort. The remaining 24 patients had no neurological symptoms affecting quality of life at last follow-up (Table 5).

Thirty patients (100%) underwent gross-total resection (GTR), and no patient underwent subtotal resection. Gross-total resection was determined based on intraoperative microscopic assessment confirming complete lesion removal and by comparing preoperative imaging with postoperative computed tomography (CT) and magnetic resonance imaging (MRI) obtained within the first 24 h after surgery, with no evidence of residual lesion on postoperative imaging.

Postoperative infection occurred in one patient. No patient required permanent shunt placement after cyst resection. The median follow-up duration was 7.0 years (IQR, 6.0–8.75 years; range, 5–10 years). No recurrence was documented during the follow-up period.

Overall, the surgical cohort demonstrated significant postoperative functional improvement (KPS), no permanent shunt dependency, and no documented recurrence during available follow-up, despite more than half of the patients presenting with radiological hydrocephalus.

One patient, a woman, experienced sudden neurological deterioration following vaginal delivery and subsequently underwent an emergency cesarean delivery. Following this acute deterioration in consciousness, cranial MRI revealed a third-ventricular colloid cyst. The patient had a preoperative Glasgow Coma Scale (GCS) score of 7 and a preoperative Karnofsky Performance Status (KPS) score of 20. Following surgical treatment, her KPS improved to 40, representing a 20-point improvement in global functional status; however, she remained bedridden in the postoperative period and had persistent motor strength deficits, as detailed in Table 5. The patient belonged to the hydrocephalus group and was not excluded from the analyses. Because the subgroup comparisons were based on medians and interquartile ranges, this single low postoperative KPS value was compatible with the median postoperative KPS of 95.0 (IQR, 90.0–100.0) in the hydrocephalus group. The lower quartile of 90.0 indicates that no more than three of the 16 patients in this group had a postoperative KPS below 90. This case illustrates that profound acute neurological deterioration before surgery may result in persistent functional deficits despite successful surgical decompression and contributes to the clinical heterogeneity of the hydrocephalus group.

## 4. Discussion

In this single-center retrospective series, microscope-assisted interhemispheric transcallosal resection of third-ventricle colloid cysts was associated with significant postoperative improvement in KPS. The median KPS increased from 80 preoperatively to 90 postoperatively, with a median improvement of 20 points. More significantly, patients with radiological hydrocephalus had lower preoperative GCS and KPS scores, indicating a much inferior baseline neurological and functional condition; nevertheless, their postoperative KPS ratings were similar to those of patients without hydrocephalus. This finding is consistent with our initial hypothesis. According to this research, when prompt surgical decompression and lesion removal are accomplished, hydrocephalus in third-ventricle colloid cysts may mainly represent reversible obstructive clinical worsening. No recurrence was documented during a median follow-up of 7.0 years (IQR, 6.0–8.75 years; range, 5–10 years), and no patient required permanent shunt placement. These results are descriptive and must be interpreted in light of the retrospective, uncontrolled design and of the limitations of the KPS as the only functional outcome measure.

This interpretation has a plausible pathophysiological basis. A colloid cyst at the foramen of Monro obstructs the outflow of CSF from both lateral ventricles, either intermittently or permanently, which can cause acute hydrocephalus and sudden neurological deterioration [3,4]. The resulting ventriculomegaly and raised intracranial pressure arise from a mechanical block rather than from a primary disorder of CSF absorption or from irreversible parenchymal injury; consistent with this, nearly half of the symptomatic patients in the natural-history series of Beaumont et al. presented with obstructive hydrocephalus [1], and hydrocephalus risk is a core element of risk-stratification tools such as the CCRS [7]. Complete removal of the cyst restores CSF circulation, so that the consequences of raised intracranial pressure—headache, vomiting, and reduced consciousness—can recover. This would explain why the hydrocephalus group reached postoperative KPS values similar to those of the non-hydrocephalus group and did not require permanent CSF diversion, and it supports the concept that even patients presenting in a compromised condition may show a meaningful improvement in global functional status after definitive relief of obstruction. Two caveats should be kept in mind. First, part of the larger KPS gain in the hydrocephalus group is arithmetical: these patients started from lower scores (median 60.0 vs. 80.0) and therefore had more room to improve, whereas patients without hydrocephalus were already close to the ceiling of the scale. Second, reversibility probably depends on the severity and duration of the obstruction; the patient who presented with a GCS of 7 and remained bedridden (Section 3.4) showed that profound acute deterioration may leave permanent deficits even after successful decompression. Our data therefore suggest, but cannot prove, that timely definitive treatment preserves the potential for functional recovery.

The ongoing debate between endoscopic and microsurgical approaches must be taken into consideration when interpreting the function of microsurgical resection. Because endoscopic surgery is less invasive and may shorten operating times, hospital stays, and perioperative morbidity, it has grown in popularity [25]. However, comparative evaluations have repeatedly demonstrated that whereas endoscopic surgery typically has a more favorable early morbidity profile, microsurgical resection offers higher rates of gross-total resection and reduced recurrence [8,13]. The absence of cyst remnants and recurrence in our series is in line with this evidence. A plausible explanation is that the transcallosal route allows direct, magnified, bimanual dissection of the cyst wall from the fornices, deep veins, and choroid plexus, because incomplete removal of the cyst wall is thought to be the main cause of regrowth [13,14]. Consequently, our findings should not be taken as proof that the transcallosal method is always better. Instead, they uphold the continued usefulness of transcallosal microsurgery as a final therapeutic approach in certain patients, especially when the management of obstructive hydrocephalus, durable resection, and direct visualization are given top priority.

Our outcomes are consistent with those of large transcallosal series, which reported favorable outcomes with no surgical mortality and mainly transient morbidity [10] and, in contemporary single-center experience, good clinical recovery and low long-term morbidity when the approach is performed with careful attention to fornix preservation, venous anatomy, and limited callosotomy [11]. In our cohort, the marked postoperative KPS improvement is consistent with these reports and reinforces that transcallosal resection remains a relevant technique even in the modern endoscopic era.

A notable finding in this series was that, despite the presence of sudden-onset symptoms of increased intracranial pressure requiring EVD in four patients and radiological findings suggestive of hydrocephalus in the majority of patients, none required permanent shunt placement. This is important because permanent CSF diversion is a clinically meaningful endpoint after colloid cyst surgery. In the meta-analysis by Sheikh et al. [8], shunt dependency did not differ significantly between microsurgical and endoscopic groups, whereas Sayehmiri et al. reported higher shunting rates after microsurgical resection compared with endoscopic resection [13]. Our zero-shunt finding may reflect effective restoration of CSF flow following cyst removal. Once the foramen of Monro is unblocked, CSF can again pass from the lateral ventricles through the third ventricle to the aqueduct and the subarachnoid spaces, and the ventricular system can re-equilibrate; temporary EVD can bridge the acute phase in patients with raised intracranial pressure, and the relatively young age of our patients (mean 30.9 years) probably favored preserved CSF absorption. Conversely, shunt dependency may occur when prolonged hydrocephalus is associated with impaired CSF absorption or when postoperative hemorrhage, infection, or adhesions compromise the CSF pathways; notably, the one patient with a postoperative infection did not become shunt-dependent. No permanent shunt dependency was observed during the available follow-up period; however, this finding should be interpreted cautiously given the modest sample size and limited duration of follow-up.

Postoperative neurological events should also be interpreted with caution. In our cohort, seizures occurred in three patients, while memory impairment, motor deficit, and visual deficit were observed in one patient each. In both patients with motor and visual deficits, these findings were already present at the time of preoperative presentation and persisted without improvement postoperatively. In contrast, none of the patients had seizures as a presenting symptom before surgery. Because no patient had a documented preoperative seizure history, the three postoperative seizures were considered new-onset events. However, their precise etiology could not be established retrospectively. The new-onset seizures may have been related to the surgical procedure itself, although a causal relationship could not be established retrospectively: the interhemispheric approach requires retraction of the medial frontal lobe and manipulation of bridging veins, with a risk of cortical contusion, venous congestion, or infarction; the callosotomy and intraventricular manipulation transgress white matter and ependymal surfaces; and ventricular catheter tracts or intraventricular blood products may add further epileptogenic stimuli. Transcallosal surgery therefore carries an inherent epileptogenic risk, and seizures are a recognized complication of this approach [10,11,18]. The timing of onset, electroencephalographic and imaging correlates, and antiepileptic prophylaxis could not be analyzed systematically in this retrospective series. These findings are within the spectrum of neurological complications reported after colloid cyst surgery, including seizures, memory impairment, venous injury or infarction, infection, hemiparesis, and other neurological deficits [18]. Transcallosal surgery in particular requires meticulous handling of the fornices, septal veins, thalamostriate veins, choroid plexus, and callosal fibers. Damage to the fornix has been reported to cause anterograde amnesia [26], and the mechanism of the memory impairment in our single affected patient cannot be determined retrospectively. All four new postoperative neurological events were persistent at last follow-up, while the two pre-existing neurological deficits also persisted. Importantly, no neurological event in this cohort resolved before last follow-up. This contrasts with series reporting mainly transient morbidity. The interpretation of these neurological events is limited by the small sample size and the presence of pre-existing deficits in two patients. These findings should therefore be interpreted in the context of the patients’ preoperative neurological status, and the designation “persistent” does not necessarily imply surgically acquired neurological injury.

The use of the KPS as the only functional outcome measure also deserves emphasis. The KPS is a global, ordinal, clinician-rated score that mainly reflects physical performance and the ability to work and care for oneself [21,22]. It has a ceiling effect, since scores of 90–100 are compatible with normal daily activities, and it is insensitive to mild or domain-specific neurocognitive deficits—such as short-term memory impairment, executive dysfunction, or disconnection phenomena—that are the typical concerns after transcallosal surgery. In our series, 13.3% of patients had new persistent neurological events (seizures in 10.0% and memory impairment in 3.3%), and subtler cognitive deficits may have gone undetected in patients with high KPS scores. We therefore use the term “functional recovery” only in the narrow sense of improvement in global performance status, and our results should not be interpreted as evidence of preserved neurocognitive function. Standardized pre- and postoperative neuropsychological testing would be required to quantify this aspect of morbidity.

Careful framing of the recurrence finding is also necessary. The current group showed no recurrence, which is reassuring and in line with the normally low recurrence rates following total microsurgical excision. Microsurgical resection has repeatedly been associated with lower recurrence compared with endoscopic resection in systematic reviews, most likely because of higher complete cyst wall removal rates [13]. In our cohort, no recurrence was documented during a median follow-up of 7.0 years (IQR, 6.0–8.75 years; range, 5–10 years). The absence of recurrence should be interpreted in the context of the extent of resection and the duration of follow-up. The long-term follow-up period and postoperative imaging assessment support durable cyst control in this series. However, given the small sample size and retrospective design, these findings should be considered descriptive and should not be interpreted as evidence that recurrence cannot occur following microsurgical resection of colloid cysts.

The radiological profile of the cohort also deserves mention, although it should not be overinterpreted. More than half of the patients had hydrocephalus, most lesions were T2 hypointense relative to CSF, most were FLAIR hyperintense, and CT hyperdensity was common. These findings are compatible with the heterogeneous imaging appearance of colloid cysts and reinforce the importance of combining imaging features with clinical presentation rather than relying on cyst size alone. The near-significant trend of larger cyst size in patients with hydrocephalus supports a biologically plausible relationship between lesion burden and obstruction, but the sample size limits statistical power. The CCRS literature similarly supports the concept that clinical and radiological variables together are more informative than any single feature alone [7].

## 5. Limitations

There are several limitations to this study. First, its single-center and retrospective design limits the generalizability of the findings and introduces the potential for selection bias. Second, the small sample size makes the subgroup analyses exploratory and precludes reliable multivariable modeling. Third, because the study did not include an endoscopic or transcortical comparison group, no conclusions regarding the superiority of the transcallosal microsurgical approach over alternative techniques can be drawn. Fourth, standardized neurocognitive assessments, including memory and executive function tests, were not performed and the KPS is insensitive to subtle cognitive deficits, limiting our ability to evaluate the potential neurocognitive effects of transcallosal surgery. Fifth, although the follow-up duration was adequate, longer-term follow-up would allow more robust assessment of the durability of resection and the risk of recurrence. Sixth, clinical and radiological data were collected retrospectively from medical records. Preoperative and postoperative imaging studies were reviewed by the surgical team, with radiological findings also evaluated based on the reports provided by the radiologist. Anesthetic variables, perioperative antiepileptic prophylaxis, and the timing of seizure onset were not analyzed. Finally, a single patient with severe preoperative neurological deterioration (GCS 7) remained bedridden postoperatively and contributed to the clinical heterogeneity of the hydrocephalus group.

Future prospective, multicenter studies should confirm and extend these findings, with standardized pre- and postoperative neuropsychological assessment (memory and executive function), validated quality-of-life measures, systematic recording of seizure characteristics and antiepileptic management, blinded independent radiological review, and longer follow-up, ideally including a matched endoscopic comparison group.

## 6. Conclusions

In this retrospective series of 30 patients, microscope-assisted interhemispheric transcallosal resection of third-ventricle colloid cysts achieved gross-total resection in all patients and was associated with a significant improvement in KPS—including in patients with preoperative hydrocephalus—with no permanent shunt requirement and no documented recurrence at a median follow-up of 7.0 years. However, 13.3% of patients developed new persistent neurological events (seizures and memory impairment), and neurocognitive outcomes were not assessed with standardized tests. Although the growing popularity of endoscopic surgical techniques and their success rates have approached those of microsurgery, microsurgical approaches continue to offer important advantages such as direct surgical visualization, the ability to perform bimanual dissection, and the potential for more radical resection. Our findings support transcallosal microsurgery as a reliable and effective option in appropriately selected patients, but they do not demonstrate superiority over endoscopic surgery. Therefore, despite the increasing use of endoscopic techniques, the advantages provided by microsurgical approaches should continue to be taken into consideration.

## Figures and Tables

**Table 1:** Variables collected and time points of assessment.

Domain	Variables	Source	Time Point
Demographic and clinical	Age, sex, presenting symptoms, symptom duration, GCS, KPS	Institutional records; admission examination	Preoperative (at admission)
Baseline radiology	Cyst diameter, hydrocephalus, T1/T2/FLAIR signal, contrast enhancement, CT density	Preoperative CT and MRI	Preoperative
Perioperative	EVD, infection, histopathology	Operative notes; inpatient and pathology records	Perioperative and early postoperative period
Early postoperative imaging	Extent of resection (gross-total resection)	CT; contrast-enhanced MRI	CT within 1 h; MRI within 24 h
Follow-up imaging	Recurrence	MRI	1, 3, and 6 months; 1 year; then at least annually
Functional status	Postoperative KPS, KPS improvement, headache status	Clinical follow-up records	last follow-up.
Neurological events	Seizure, memory impairment, motor deficit, visual deficit (new vs. pre-existing; transient vs. persistent)	Clinical follow-up records	Persistence: last follow-up. Onset: postoperative period
Shunt requirement	Permanent shunt placement	Clinical follow-up records	Entire long-term follow-up

**Table 2:** Baseline demographic, clinical, and radiological features of the study cohort. Continuous variables are presented as mean ± SD and median (IQR); categorical variables as *n* (%).

Characteristic	Value
Age, years	30.9 ± 10.6; median 31.0 (24.2–38.0)
Female sex	17 (56.7)
Male sex	13 (43.3)
Cyst size, mm	14.0 ± 6.0; median 12.5 (10.0–17.5)
Radiological hydrocephalus	16 (53.3)
Preoperative GCS	14.2 ± 1.9; median 15.0 (14.0–15.0)
Preoperative KPS	65.3 ± 21.0; median 80.0 (42.5–80.0)
Postoperative KPS	92.0 ± 11.0; median 90.0 (90.0–100.0)
KPS improvement	26.7 ± 19.7; median 20.0 (10.0–37.5)
T1 hypointense	18 (60.0)
T1 hyperintense	12 (40.0)
T2 hypointense relative to CSF	29 (96.7)
FLAIR hyperintense	23 (76.7)
Contrast enhancement	0 (0.0)
CT hyperdense	20 (66.7)
CT isodense	8 (26.7)
CT hypodense	2 (6.7)

**Table 3:** Perioperative management, postoperative complications, and functional outcomes after transcallosal microsurgical resection.

Characteristic	*n* (%) or Value
External ventricular drainage	4 (13.3)
Permanent shunt requirement	0 (0.0)
Postoperative infection	1 (3.3)
New postoperative neurological event	4 (13.3)
Pre-existing neurological deficit persisting postoperatively	2 (6.7)
Postoperative seizure (new)	3 (10.0)
Postoperative memory impairment (new)	1 (3.3)
Persistent motor deficit (pre-existing)	1 (3.3)
Persistent visual deficit (pre-existing)	1 (3.3)
Follow-up duration, years	median (IQR), range: 7.0 (6.0–8.75), 5–10
Recurrence	0 (0.0)
Postoperative KPS	92.0 ± 11.0; median 90.0 (90.0–100.0)
KPS improvement	26.7 ± 19.7; median 20.0 (10.0–37.5)
Gross-total resection	30 (100.0)
Operative approach	Interhemispheric transcallosal (all cases)

**Table 4:** Comparison of clinical characteristics and postoperative outcomes between patients with and without preoperative hydrocephalus. Data are median (IQR) unless otherwise specified; *p* values were obtained with the Mann–Whitney U test (continuous variables) or Fisher’s exact test (categorical variables).

Variable	No Hydrocephalus (*n* = 14)	Hydrocephalus (*n* = 16)	*p* Value
Age, years	33.1 ± 10.2; median 34.5 (27.2–38.8)	28.9 ± 11.0; median 30.5 (21.0–34.2)	0.279
Cyst size, mm	11.0 (10.0–13.5)	15.0 (11.0–20.0)	0.076
Preoperative GCS	15.0 (15.0–15.0)	14.5 (14.0–15.0)	0.022
Preoperative KPS	80.0 (80.0–80.0)	60.0 (40.0–80.0)	0.048
Postoperative KPS	90.0 (90.0–90.0)	95.0 (90.0–100.0)	0.208
KPS improvement	10.0 (10.0–20.0)	20.0 (20.0–50.0)	0.012
EVD requirement, *n* (%)	0 (0.0)	4 (25.0)	0.103
Any neurological event or persistent deficit, *n* (%)	2 (14.3)	4 (25.0)	0.657
Infection, *n* (%)	0 (0.0)	1 (6.3)	1.000

**Table 5:** Neurological events and persistent neurological deficits: type, onset, and status at last follow-up.

Neurological Event	*n* (%)	Onset	Status at Last Follow-Up
Seizure	3 (10.0)	New postoperative	Persistent—controlled with antiepileptic therapy
Memory impairment	1 (3.3)	New postoperative	Persistent—interfering with daily activities
Motor deficit	1 (3.3)	Preoperative (not resolved)	Persistent—present since preoperative deterioration
Visual deficit	1 (3.3)	Preoperative (not resolved)	Persistent—present since preoperative deterioration
Any neurological event or persistent deficit	6 (20.0)	—	All persistent; none transient

## Data Availability

The data presented in this study are available from the corresponding author upon reasonable request and subject to institutional and ethical restrictions regarding patient privacy.
